# Epidemiological, Radiographical, and Laboratorial Characteristics of Chinese Asymptomatic Cases With COVID-19: A Systematic Review and Meta-Analysis

**DOI:** 10.3389/fpubh.2022.808471

**Published:** 2022-03-31

**Authors:** Haohao Yan, Yudan Ding, Wenbin Guo

**Affiliations:** ^1^Department of Psychiatry, National Clinical Research Center for Mental Disorders, The Second Xiangya Hospital of Central South University, Changsha, China; ^2^Department of Psychiatry, The Third People's Hospital of Foshan, Foshan, China

**Keywords:** COVID-19, SARS-CoV-2, asymptomatic infection, presymptomatic infection, CT

## Abstract

The comprehensive understanding of the characteristics of asymptomatic cases are helpful for the identification and management of patients with asymptomatic COVID-19 infection. Four electronic databases were searched from December 1, 2019 to February 8, 2022 for relevant articles. Data synthesis, subgroup analysis, and sensitivity analysis were performed on the included studies. *I*^2^ and Q tests were applied to evaluate heterogeneity across studies. The risk of publication bias was assessed and visualized using a funnel plot. A total of 45 studies consisting of 2,655 patients with no symptoms at the screening point were included. Pooled results showed that in China, 65% of initial no-symptoms COVID-19 patients did not present any COVID-19-related symptom during follow-up or by end of disease course (asymptomatic infections). High proportions of initial no-symptoms COVID-19 patients (76%) and patients with asymptomatic infection (55%) had abnormal CT features at the screening point. High proportion of patients with asymptomatic infection had been detected Ig G^+^ (72%) and/or Ig M^+^ (57%) at the screening point. The chest CT scan and SARS-CoV-2-specific antibody testing could serve as effective supplementary methods to identify asymptomatic cases in the early stage of SARS-CoV-2 infection. However, the chest CT scan and the SARS-CoV-2-specific IgM and IgG testing should not replace reverse transcription–polymerase chain reaction (RT-PCR) for screening in asymptomatic patients. The combination of repeated RT-PCR, chest CT scans, and the SARS-CoV-2-specific IgM and IgG testing should be performed for those highly suspected SARS-CoV-2 infections.

**Systematic Review Registration:**

https://www.crd.york.ac.uk/PROSPERO/#recordDetails, identifier: CRD 42021261130.

## Introduction

The rapid spread of the coronavirus disease 2019 (COVID-19) epidemic has caused an ongoing global pandemic due to the highly contagious severe acute respiratory syndrome coronavirus 2 (SARS-CoV-2) and the susceptibility of people. The diagnosis of COVID-19 is based on epidemiological history, symptoms, virus nucleic acid detection, imaging, and laboratory results according to the Chinese Guidelines for the Diagnosis and Treatment Plan of SARS-CoV-2 Infection by the National Health Commission (Trial Version 5) (1). At present, upper respiratory tract nasopharyngeal swabs are mostly used as nucleic acid detection samples in China, but the positive rate is low (2). Although multi-sample 2019-nCoV nucleic acid detection can improve the accuracy and reduce the false negative rate, the cost is high (2, 3). Given the high cost of nucleic acid testing, shortage of medical supplies, and rapid increase in the number of infections, some countries monitor the temperature to screen suspected infections for isolation and management. However, many asymptomatic cases have been reported. Studies showed that asymptomatic cases may account for about 60% of all patients with COVID-19, and viral replication in these cases was robust, and the virus was highly infectious (4–8). Asymptomatic cases have a similar viral load as symptomatic cases (9). A mathematical model incorporating asymptomatic cases indicates that asymptomatic cases are major drivers for the growth of the COVID-19 pandemic (10). Most asymptomatic patients do not seek medical assistance due to no obvious clinical symptoms and poor prevention awareness, which contribute to the rapid spread of COVID-19. Although secondary attack rate may be 3–25 times lower for asymptomatic patients than for those with symptoms, the high proportion in total infections and difficulty in identification make asymptomatic cases as major drivers for COVID-19 pandemic (11, 12). The early recognition of infections and cutting off the route of transmission are key points to control the COVID-19 pandemic. However, we can only rely on immunology testing, radiographical scan or nucleic acid detection technology to obtain information about asymptomatic infections. Therefore, this kind of infectious source cannot be effectively identified, making it very difficult to be controlled and prevented. Considering that asymptomatic cases are more difficult to identify than symptomatic cases, control interventions may be undermined. In China, the COVID-19 pandemic has been gradually controlled. At present, the identification and management of patients with asymptomatic infection has become an urgent problem that needs to be addressed. The comprehensive understanding of the epidemiological, radiographical, and laboratorial characteristics of asymptomatic cases are helpful for the identification and management of patients with asymptomatic infection. To comprehensive understanding of these characteristics of asymptomatic cases, the present systematic review and meta-analysis is performed.

## Materials and Methods

A meta-analysis was conducted in accordance with the Preferred Reporting Items for Systematic Reviews and Meta-analyses (PRISMA) (13) and Meta-Analysis of Observational Studies in Epidemiology guidelines (14). The review protocol was registered at PROSPERO as CRD 42021261130.

### Search Strategy

Two authors (YD and HY) independently identified relevant articles published in Embase, PubMed, China National Knowledge Infrastructure, and WANFANG DATA from December 1, 2019 to February 8, 2022. We applied the following terms in retrieving studies from the PubMed database: (COVID-19 OR SARS-CoV-2 OR 2019-nCoV Disease) AND (asymptomatic OR pre-symptomatic OR covert infection). Modifications were made as required to retrieve studies from other electronic databases. The search strategy had also been provided in PROSPERO. Besides, the reference lists of the included studies were hand-searched to acquire additional relevant articles.

### Study Selection Criteria

Initially no-symptom patients were those with presymptomatic or asymptomatic infection at the screening point. Initial no-symptoms COVID-19 patients were defined as individuals who were positive for SARS-CoV-2, detected by reverse transcription–polymerase chain reaction (RT-PCR), but had no COVID-19-related clinical symptom at the screening point. Presymptomatic infections or patients with presymptomatic infection were defined as individuals who had no symptoms at the diagnosis time but presented COVID-19-related symptoms during follow-up. Asymptomatic infections or patients with asymptomatic infection were defined as individuals who did not present any COVID-19-related symptom during follow-up or by end of disease course but had a positive result of RT-PCR at the screening point.

The inclusion criteria were as follows: (i) the participants were Chinese who had asymptomatic COVID-19 infection at the screening point; (ii) studies that reported data about the number of patients with presymptomatic or asymptomatic infection and (iii) radiographical or laboratorial characteristics of asymptomatic patients.

The exclusion criteria were as follows: (i) duplicate publication data; (ii) case reports, reviews, commentaries, and conference abstracts; and (iii) studies in which the number of participants <10 were excluded.

### Data Extraction and Quality Assessment

Two authors (YD and HY) independently extracted the following data from the included articles: name of the first author, participants, study design, location, time of data collection, sample size, age, number of males, method to determine an infection, time of performing a chest computerized tomography (CT) scan, duration of viral shedding, duration of symptoms developed, and radiographical and laboratorial results.

An 11**-**item checklist recommended by the Agency for Healthcare Research and Quality was applied to assess the quality of included studies (15, 16). If an item was answered “NO” or “UNCLEAR” it would be scored 0 and if it was answered “YES,” then the item scored 1. The studies were categorized into low (0–3), moderate (4–7) and high quality (8–11).

During data extraction and quality assessment, a third team member (WG) performed verification. All discrepancies were discussed and resolved by the three authors.

### Data Analysis

Data analysis was performed using the Stata software version 14.0 (Stata Corp. LP, College Station, USA). For the anticipated clinical heterogeneity, the pooled proportions of patients with asymptomatic infection in the initially no-symptoms COVID-19 patients, individuals with abnormal CT features in the initially no-symptoms COVID-19 patients at the screening point, individuals with abnormal CT features in patients with asymptomatic infection at the screening point, bilateral lung involvement in the initially no-symptoms COVID-19 patients with abnormal CT features at the screening point, bilateral lung involvement in asymptomatic infections with abnormal CT features at the screening point, IgM^+^ or IgG^+^ in patients with asymptomatic infection at the screening point with 95% confidence interval (CI) were calculated using the random-effects model. The random-effects model was considered to be suitable for meta-analyses with substantial heterogeneity. We performed the Freeman–Tukey double arcsine transformation before data pooling due to some included studies that reported these proportions close to 1 or 0. *I*^2^ (significance level of *I*^2^ > 50 %) and Q tests (significance level of *p* < 0.05) were applied to evaluate heterogeneity across studies. A sensitivity analysis was conducted to evaluate the robustness and reliability of the pooled proportions. Subgroup analysis was performed according to location: Hubei Province or outside of Hubei Province (Wuhan is located in Hubei Province), and sample size (more than or not more than 30 participants) to explore the potential source of heterogeneity. The risk of publication bias was assessed and visualized using a funnel plot.

## Results

### Literature Search

**Figure 1:**
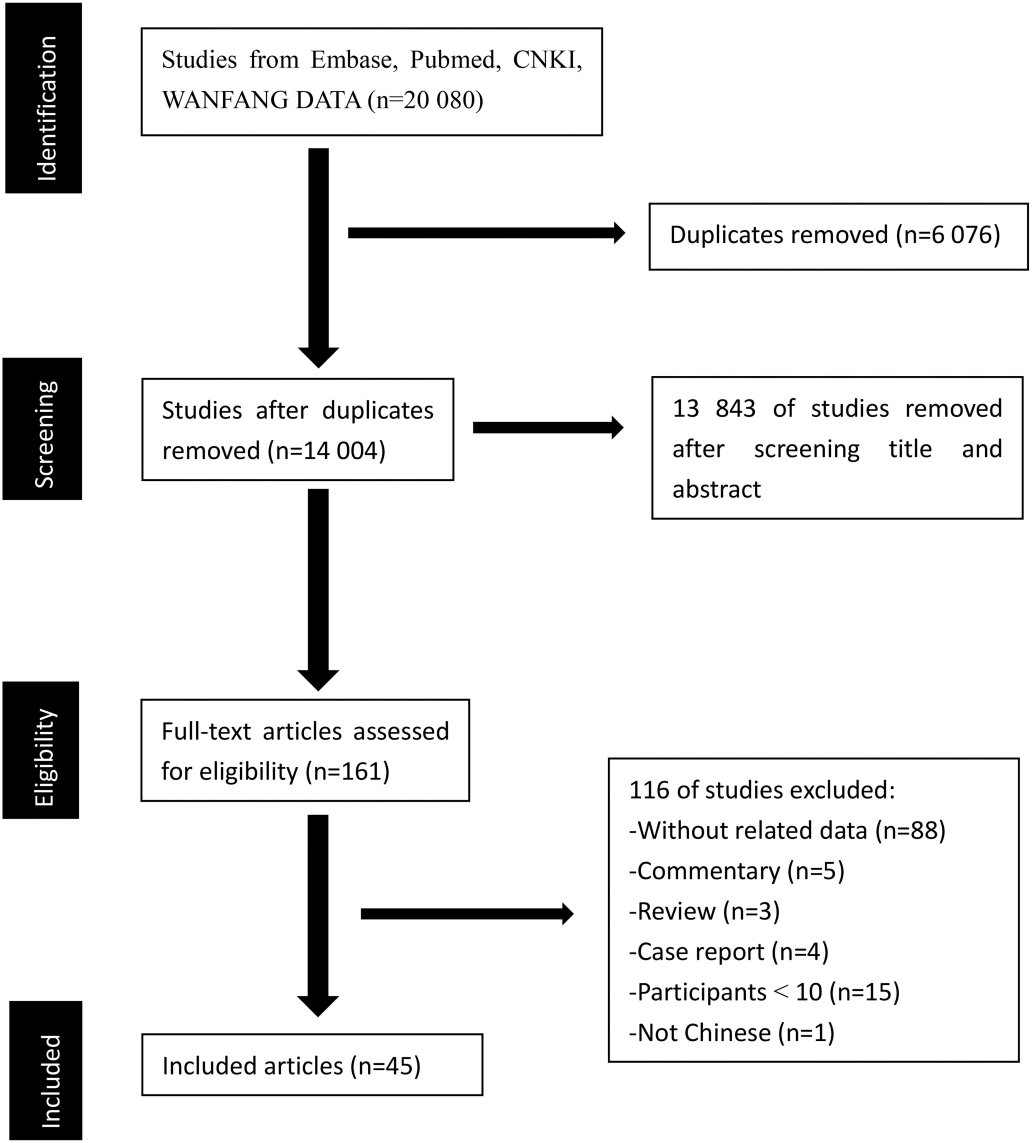
PRISMA diagram of the article selection.

Our initial search identified 20 080 records (7,559, 11,025, 624, and 872 records in Pubmed, Embase, China National Knowledge Infrastructure, and WANFANG DATA, respectively). A total of 6,076 articles were duplicates. After duplicates were removed, 13,843 studies were excluded after reviewing titles and abstracts. A total of 161 potentially relevant records were retrieved for detailed full-text evaluation. Finally, 45 articles (17–61) met the selection criteria and were deemed to have relevant data to the meta-analysis. A PRISMA diagram detailing the process of article selection was shown in Figure 1.

### Characteristics of Included Studies

A total of 45 studies consisting of 2,655 patients with no symptoms at the screening point were included in the meta-analysis, among which 13 articles were Chinese articles (18–20, 24, 34, 35, 37, 41, 46, 53, 58–60) and 32 articles were English articles. Six studies (26, 48, 52, 56–58) were prospective studies and the rest were retrospective studies. The number of studies in which the participants came from Wuhan was the largest (11/41). The participants were initial no-symptoms COVID-19 patients in 22 studies. The other 23 studies involved participants with asymptomatic infection. All participants were children in two studies (55, 56). The proportion of males was 48.1%. The time of data collection of most studies (28/37) was between January and April 2020. Eight studies did not report the time of data collection. All studies applied nucleic acid testing to determine a diagnosis. In all included studies, the CT scan and laboratorial sampling were performed on admission or to determine a diagnosis (the asymptomatic phase). Ten studies reported the duration of viral shedding. Ten studies reported the duration of COVID-19-related symptoms developed. A summary of characteristics of 45 included studies was shown in Supplementary Table 1 in the supplementary materials.

The result of quality assessment was shown in Supplementary Table 2. All studies were of high (5/45) or moderate (40/45) quality.

### Meta-Analysis Results

#### Proportion of Patients With Asymptomatic Infection in Initial No-Symptoms COVID-19 Patients

The proportion of patients with asymptomatic infection in initial no-symptoms COVID-19 patients was 65% (95% CI: 58–72%, *I*^2^ = 88.4%, k = 22, *n* = 1,769; Supplementary Figure 1) (18, 19, 22, 24, 26–30, 34, 35, 39, 41, 43, 44, 46, 48, 52–54, 57, 61). In the sensitivity analysis, we found no study that affected the proportion by over 3%.

#### Proportion of Individuals With Abnormal CT Features in Initial No-Symptoms COVID-19 Patients at the Screening Point

**Figure 2:**
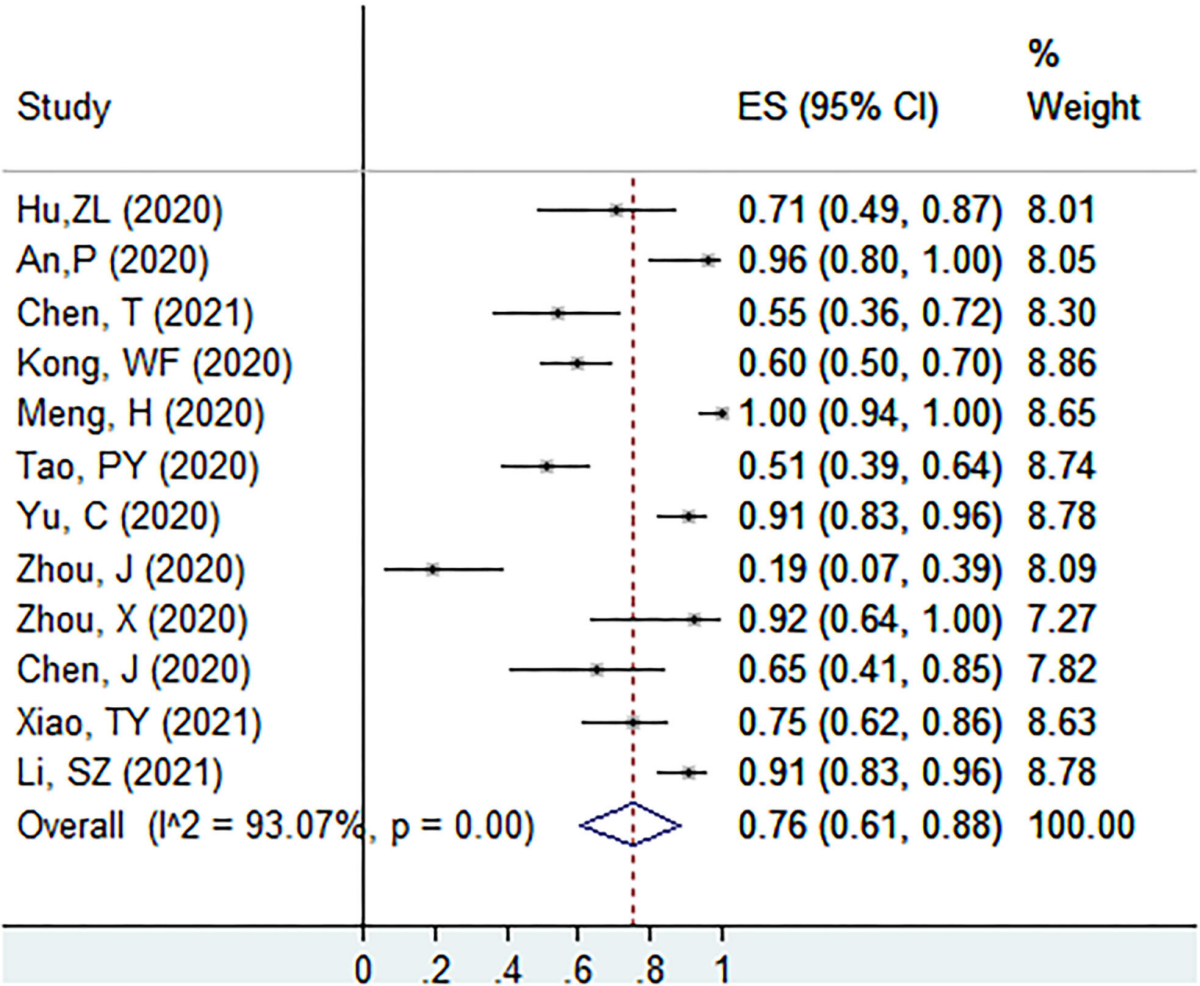
The proportion of individuals with abnormal CT features in initial no-symptoms COVID-19 patients at the screening point.

The proportion of individuals with abnormal CT features in initial no-symptoms COVID-19 patients at the screening point was 76% (95% CI: 61–88%, *I*^2^ = 93.1%, k = 12, *n* = 583; Figure 2) (22, 27–30, 39, 43, 52, 54, 55, 57, 61). In the sensitivity analysis, we found two studies that affected the proportion by over 3%.

#### Proportion of Individuals With Abnormal CT Features in Patients With Asymptomatic Infection at the Screening Point

**Figure 3:**
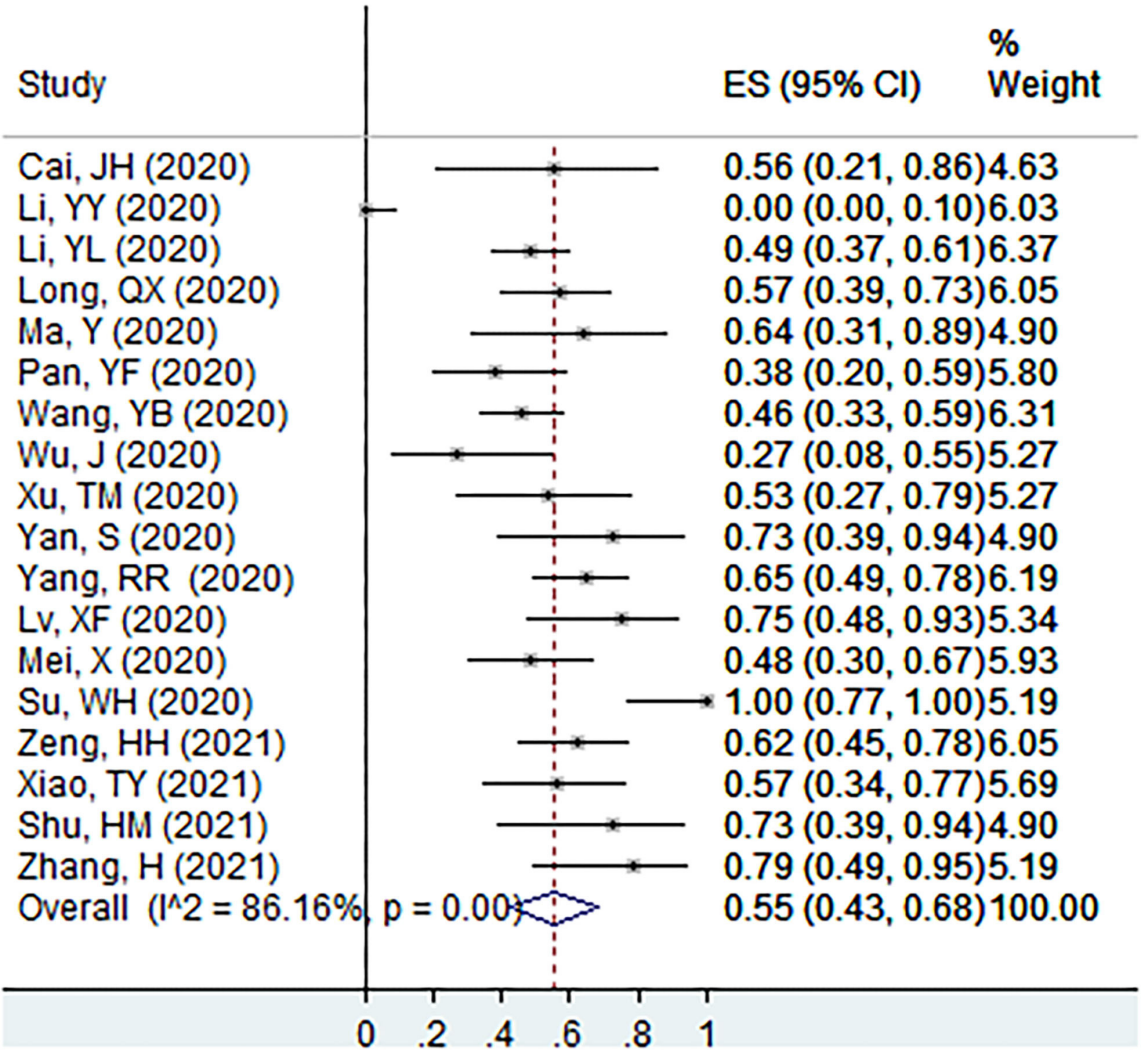
The proportion of individuals with abnormal CT features in patients with asymptomatic infection at the screening point.

The proportion of individuals with abnormal CT features in patients with asymptomatic infection at the screening point was 55% (95% CI: 43–68%, *I*^2^ = 86.2%, k = 18, *n* = 491; Figure 3) (20–23, 31–33, 36, 38, 42, 44–47, 49, 50, 56, 59). In the sensitivity analysis, we found one study that affected the proportion by over 3%.

#### Proportion of Bilateral Lung Involvement in Initial No-Symptoms COVID-19 Patients With Abnormal CT Features at the Screening Point

The proportion of bilateral lung involvement in initial no-symptoms COVID-19 patients with abnormal CT features at the screening point was 56% (95% CI: 37–74%, *I*^2^ = 87.9%, k = 5, *n* = 243; Supplementary Figure 2) (30, 39, 43, 52, 54).

#### Proportion of Bilateral Lung Involvement in Asymptomatic Infections With Abnormal CT Features at the Screening Point

The proportion of bilateral lung involvement in asymptomatic infections with abnormal CT features at the screening point was 55% (95% CI: 41–69%, *I*^2^ = 36.2%, k = 7, *n* = 95; Supplementary Figure 3) (23, 33, 42, 45, 46, 49, 59).

#### Proportion of Ig G^+^ in Patients With Asymptomatic Infection at the Screening Point

**Figure 4:**
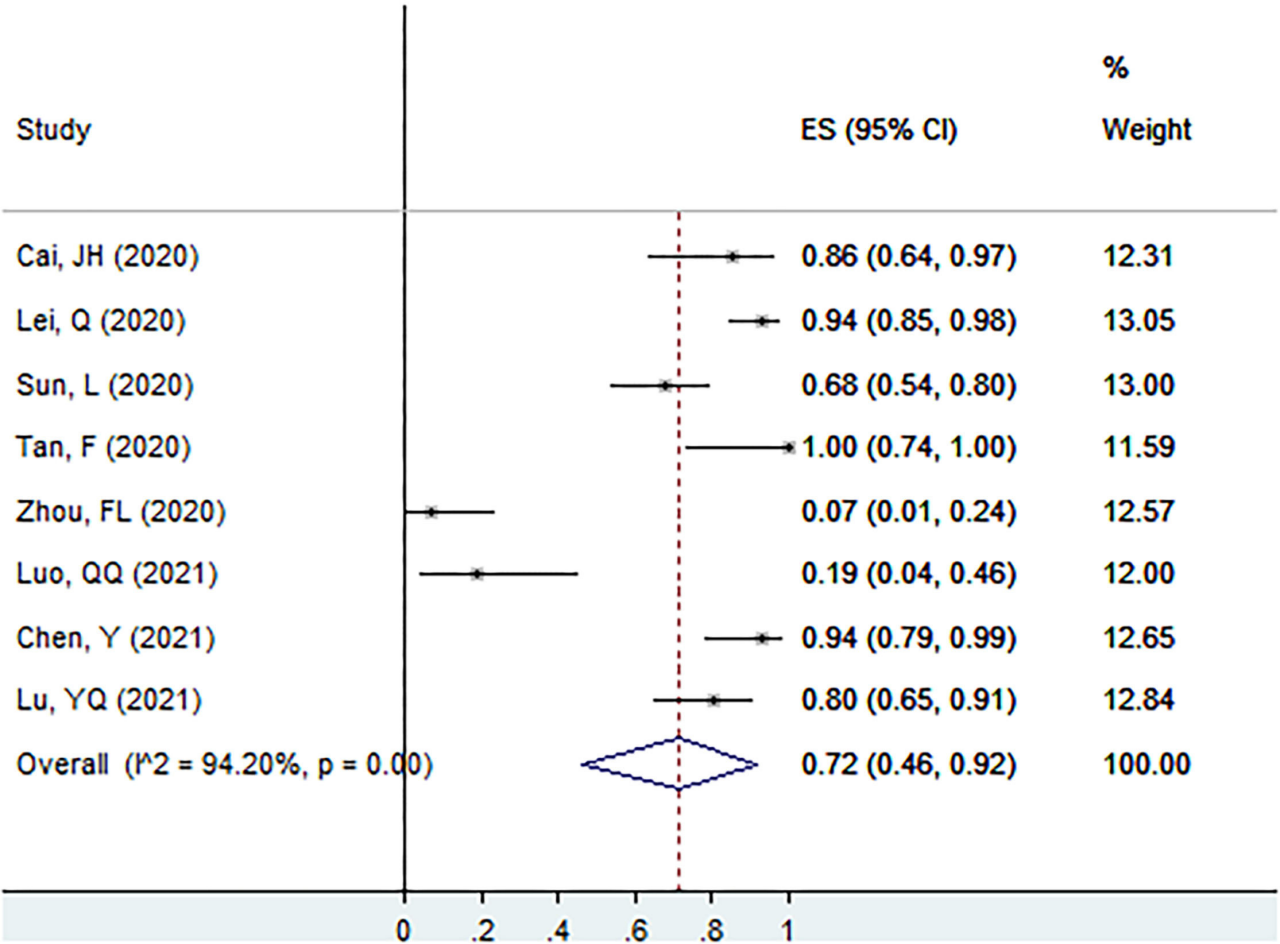
The proportion of Ig G^+^ in patients with asymptomatic infection at the screening point.

The proportion of Ig G^+^ in patients with asymptomatic infection at the screening point was 72% (95% CI: 46–92%, *I*^2^ = 94.2%, k = 8, *n* = 268; Figure 4) (17, 25, 26, 40, 51, 56, 58, 60).

#### Proportion of Ig M^+^ in Patients With Asymptomatic Infection at the Screening Point

**Figure 5:**
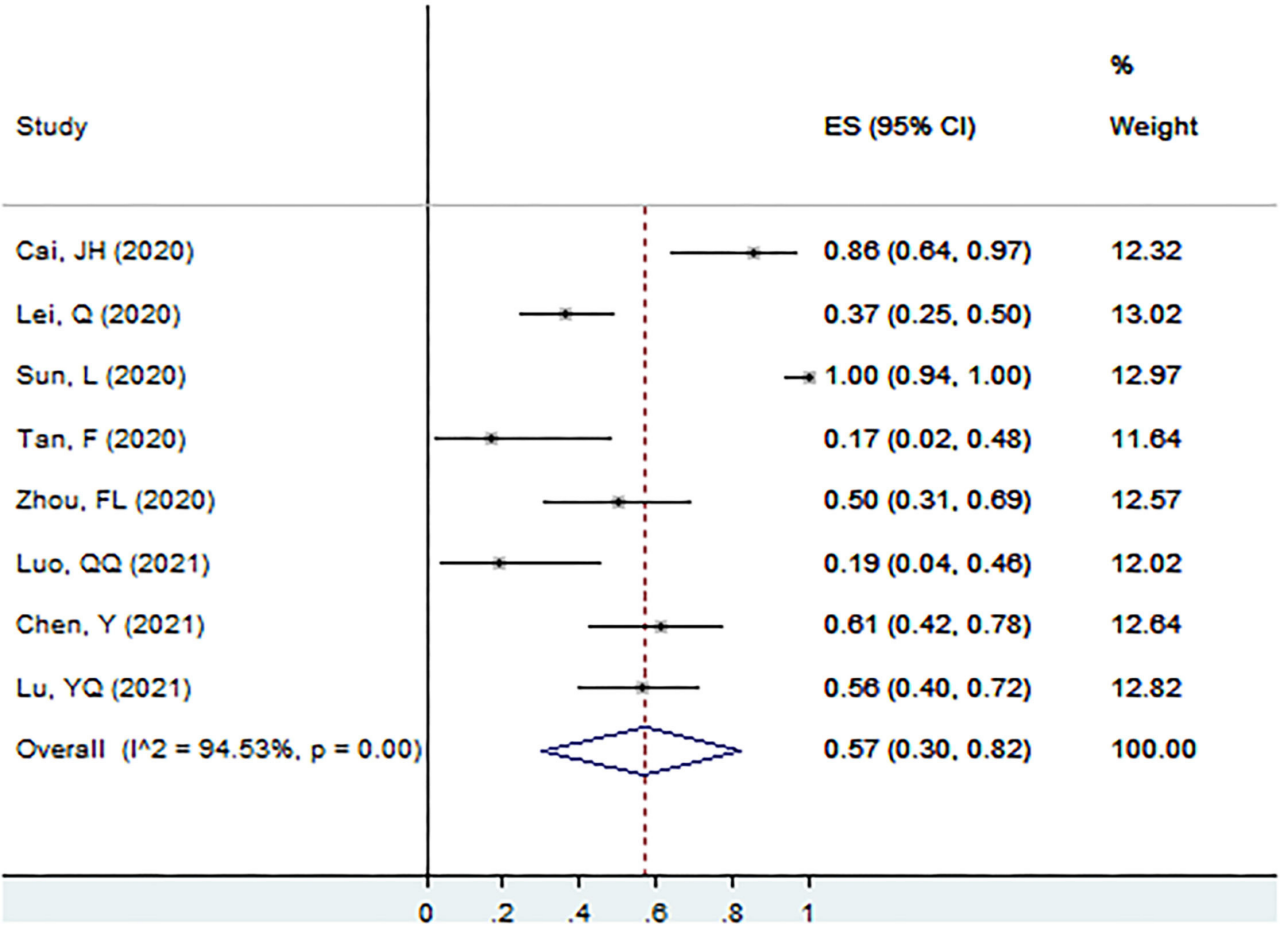
The proportion of Ig M^+^ in patients with asymptomatic infection at the screening point.

The proportion of Ig M^+^ in patients with asymptomatic infection at the screening point was 57% (95% CI: 30–82%, *I*^2^ = 94.5%, k = 8, *n* = 268; Figure 5) (17, 25, 26, 40, 51, 56, 58, 60).

### Subgroup Analysis Results

#### Hubei Province-Based Studies vs. Other Locations

The pooled proportion of patients with asymptomatic infection in initial no-symptoms COVID-19 patients in studies that used Hubei Province as a survey site (k = 5, total n = 257) was 60% (95% CI: 52–68%, *I*^2^ = 36.0%). The pooled proportion of patients with asymptomatic infection in initial no-symptoms COVID-19 patients in studies that used locations outside Hubei Province as survey sites (k = 16, total *n* = 1,486) was 67% (95% CI: 58–76%, *I*^2^ = 91.4%).

The proportion of individuals with abnormal CT features in initial no-symptoms COVID-19 patients at the screening point in studies that used Hubei Province as a survey site (k = 4, total *n* = 241) was 95% (95% CI: 89–100%, *I*^2^ = 71.0%). The proportion of individuals with abnormal CT features in initial no-symptoms COVID-19 patients at the screening point in studies that used locations outside Hubei Province as survey sites (k = 7, total *n* = 316) was 65% (95% CI: 56–74%, *I*^2^ = 60.3%).

The proportion of individuals with abnormal CT features in patients with asymptomatic infection at the screening point in studies that used Hubei Province as a survey site (k = 6, total *n* = 219) was 55% (95% CI: 24–84%, *I*^2^ = 95.2%). The proportion of individuals with abnormal CT features in patients with asymptomatic infection at the screening point in studies that used locations outside Hubei Province as survey sites (k = 12, total *n* = 272) was 55% (95% CI: 47–63%, *I*^2^ = 32.0%).

#### Studies Had More Than 30 Participants vs. Not More Than 30 Participants

The pooled proportion of patients with asymptomatic infection in initial no-symptoms COVID-19 patients in studies that had more than 30 participants (k = 15, total *n* = 1,625) was 64% (95% CI: 55–72%, *I*^2^ = 91.5%). The pooled proportion of patients with asymptomatic infection in initial no-symptoms COVID-19 patients in studies that had not more than 30 participants (k = 7, total *n* = 144) was 71% (95% CI: 59–82%, *I*^2^ = 51.3%).

The proportion of individuals with abnormal CT features in initial no-symptoms COVID-19 patients at the screening point in studies that had more than 30 participants (k = 7, total *n* = 475) was 79% (95% CI: 61–92%, *I*^2^ = 94.5%). The proportion of individuals with abnormal CT features in initial no-symptoms COVID-19 patients at the screening point in studies that had not more than 30 participants (k = 5, total *n* = 108) was 71% (95% CI: 39–95%, *I*^2^ = 91.0%).

The proportion of individuals with abnormal CT features in patients with asymptomatic infection at the screening point in studies that had more than 30 participants (k = 7, total *n* = 326) was 44% (95% CI: 25–63%, *I*^2^ = 92.2%). The proportion of individuals with abnormal CT features in patients with asymptomatic infection at the screening point in studies that had not more than 30 participants (k = 11, total *n* = 165) was 64% (95% CI: 49–78%, *I*^2^ = 70.7%).

#### Other Laboratorial Characteristics

Laboratorial characteristics were shown in Table 1. Eleven of the included studies reported abnormal lymphocyte count at the screening point. A total of 5 studies with 27 out of 177 (15.3%) initial no-symptoms COVID-19 patients and 5 studies with 21 out of 140 (15.0%) patients with asymptomatic infection reported lymphocytopenia. One study with patients with asymptomatic infection reported lymphocytosis (4/11, 36.4%). Sixteen of the included studies reported abnormal white blood cell count at the screening point. A total of 5 studies with 13 out of 138 (9.4%) initial no-symptoms COVID-19 patients and 5 studies with 15 out of 135 (11.1%) patients with asymptomatic infection reported leukopenia. A total of 4 studies with 11 out of 121 (9.1%) initial no-symptoms COVID-19 patients and 3 studies with 3 out of 41 (7.3%) asymptomatic infections reported leukocytosis. Elevated C-reactive protein (CRP) values (Six studies), procalcitonin (PCT) (2 studies), lactate dehydrogenase (LDH) (6 studies), alanine aminotransferase (ALT) (4 studies), Creatinine (Cr) (4 studies), D-dimer levels (4 studies), erythrocyte sedimentation rate (ESR) (5 studies), and reduced albumin (4 studies) and hemoglobin levels (1 study) were reported at the screening point. We did not pool the proportions of these laboratorial characteristics due to limited data.

**Table 1:** Laboratorial characteristics of Chinese asymptomatic cases with COVID-19.

**Author**	**Participants**	**Sample size**	**Laboratorial characteristics**
Hu, ZL et al.	A	24	4 lymphocyte↓, 4 WBC↓, 4 CRP↑, 5 PCT↑, 4 LDH↑, 2 ALT↑, 2 Cr↑, 4 D-dimer↑
An, P et al.	A	25	NA
Cai, JH et al.	B	21	18 IgG^+^ and IgM^+^
Chen, T et al.	A	33	19 PCT↑,12 lymphocyte↓
Kong, WF et al.	A	100	NA
Lei, Q et al.	B	63	4 IgG^−^and IgM^−^, 36 IgG^+^ and IgM^−^, 23 IgG^+^ and IgM^+^
Li, YY et al.	B	38	6 ESR↑
Li, YL et al.	B	74	NA
Liu, ZR et al.	A	147	NA
Long, QX et al.	A	37	3 lymphocyte↓, 1 PLT↓, 6 ALT↑, 11 CRP↑
Ma, Y et al.	B	11	3 WBC↓,4 lymphocyte↑
Mei, X et al.	B	39	NA
Meng, H et al.	A	58	NA
Pan, YF et al.	A	26	2 WBC↓, 1 WBC↑, 4 PLT↑, 4 D-dimer↑, 3 albumin↓, 2 Cr↑, 3 LDH↑, 1 CK↑
Tan, F et al.	B	12	1 WBC↑, 1 Neutrophil↑, 2 lymphocyte↓, 3 CRP↑, 2 LDH↑, 3 D-dimer↑, 10 IgG^+^ and IgM^−^, 2 IgG^+^ and IgM^+^
Tao, PY et al.	B	70	4 WBC↓, 8 lymphocyte↓
Wang, YB et al.	A	63	4 WBC↓, 4 WBC↑, 6 lymphocyte↓
Wu, J et al.	B	15	10 ESR↑, 8 albumin↓
Xu, TM et al.	B	15	1 lymphocyte↓, 2 CRP↑, 1 Neutrophil↓, 1 PLT↓, 3 ALT↑, 6 LDH↑, 2 albumin↓, 1 Cr↑, 1 D-dimer↑
Yan, S et al.	B	11	1 WBC↑, 1 WBC↓, 1 Neutrophil↑, 1 Neutrophil↓, 1 CRP↑, 2 ESR↑
Yang, RR et al.	B	48	NA
Yu, C et al.	A	79	NA
Zhou, FL et al.	B	28	2 IgM^+^ and IgG^+^, 12 IgM^+^ and IgG^−^
Zhou, J et al.	A	26	NA
Zhou, X et al.	A	13	2 WBC↓, 10 ESR↑
Huang, XM et al.	A	198	NA
Lv, XF et al.	B	16	NA
Sun, BH et al.	A	32	NA
Wang, YF et al.	B	159	NA
Xie, SL et al.	A	325	NA
Xiong, Y et al.	A	242	NA
Chen, J et al.	A	20	5 WBC↑, 2 lymphocyte↓, 3 ESR↑, 7LDH↑, 4 lactate↑
Zeng, HH et al.	B	37	NA
Xiao, TY et al.	A	56	NA
Shu, HM et al.	B	11	NA
Luo, QQ et al.	B	16	7 WBC↑, 2 Lymphopenia, 3 IgM^+^, 3 IgG^+^
Chen, Y et al.	A	45	C: 5 IgM^+^, B: 19 IgM^+^, C: 9 IgG^+^, B: 29 IgG^+^
Ni, Z et al.	A	28	NA
Zhao BN et al.	A	12	1 WBC↑, 1 WBC↓, 3 CRP↑, 5 serum amyloid A↑, 6 CD4^+^ T cell ↓, 3 CD8^+^T cell↓, 6 NK cell↓
Zhang YN et al.	A	160	NA
Zhang H et al.	B	25	6 WBC↓, 2 Neutrophil↑, 3 lymphocyte↓
Sun L et al.	B	56	56 IgM^+^, 38 IgG^+^
Su WH et al.	B	18	1 WBC↑, 1 WBC↓, 7 Neutrophil↑, 7 lymphocyte↓, 10 hemoglobin↓, 5 ALT↑, 7 LDH↑, 2 Cr↓, 12 albumin↓
Lu YQ et al.	B	41	23 IgM^+^, 33 IgG^+^

*A, Initial no-symptoms COVID-19 patients; B, Patients with asymptomatic infection; C, Patients with pre-symptomatic infection; NA, Not available; /, Not applicable; WBC, White blood cell; CRP, C reactive protein; PCT, Procalcitonin; LDH, Lactate dehydrogenase; ALT, Alanine aminotransferase; Cr, Creatinine; PLT, platelet; CK, Creatine kinase; ESR, Erythrocyte sedimentation rate; NK cell, natural killer cell. ↑, elevated; ↓, declined*.

#### Other Features of Initial No-Symptoms COVID-19 Patients, Patients With Asymptomatic Infection, and Patients With Presymptomatic Infection

Patients with asymptomatic infection were significantly younger than patients with presymptomatic infection (26, 27, 35, 53). Initially no-symptom COVID-19 patients or patients with asymptomatic infection were significantly younger than symptomatic patients (44, 47). Asymptomatic infections with normal chest CT scans were significantly younger than asymptomatic infections with abnormal chest CT scans (21, 56).

The viral shedding time of asymptomatic infections was significantly shorter than that of patients with presymptomatic infection (22, 26, 27). The viral shedding time of asymptomatic infections with normal chest CT scans was significantly shorter than asymptomatic infections with abnormal chest CT scans (56). The viral shedding time of initially no-symptom patients with normal chest CT scans was significantly shorter than that of initially no-symptom patients with abnormal chest CT scans (54).

The levels of virus-specific IgG in the asymptomatic infections were significantly lower than those of the symptomatic patients in the acute phase (47). The positive rate of IgM antibody testing was significantly lower in asymptomatic infections than that in symptomatic patients during follow-up (25).

Some studies reported the improvement or further radiological progress of chest CT scans in initially no-symptom patients (27, 42, 47, 57). Other studies reported that in initially no-symptom patients (54, 57) or asymptomatic infections (27, 31, 42, 56) without any radiological findings at the time of diagnosis, no radiological findings were observed on the follow-up CT.

Liu, ZR et al. found that the second attack rate in patients with presymptomatic infection was 9.7% and the second attack rate in asymptomatic infections was 2.6% (48). Other included studies reported the asymptomatic infections were infectious (34, 53).

#### Publication Bias

The funnel plots of the proportion of patients with asymptomatic infection in initial no-symptoms COVID-19 patients, proportion of individuals with abnormal CT features in initial no-symptoms COVID-19 patients at the screening point, and proportion of individuals with abnormal CT features in patients with asymptomatic infection at the screening point were shown in Supplementary Figures 4–6, respectively. No evident publication bias was detected. The funnel plots of other domains were not exhibited due to limited data.

## Discussion

A total of 45 studies consisting of 2,655 patients with no symptoms at the screening point were included in the systematic review and meta-analysis. Pooled results showed that in China, 65% of initial no-symptoms COVID-19 patients did not present any COVID-19-related symptom during follow-up or by end of disease course (asymptomatic infections). High proportions of initial no-symptoms COVID-19 patients and patients with asymptomatic infection had abnormal CT features at the screening point. Near half of initial no-symptoms COVID-19 patients and asymptomatic infections with abnormal CT features had bilateral lung abnormality. High proportion of patients with asymptomatic infection had been detected Ig G^+^ and/or Ig M^+^ at the screening point.

We found that in most of the included studies, the median duration of viral shedding in patients with asymptomatic infection was shorter than 15 days, which was shorter than that of symptomatic patients (9, 62–64). A meta-analysis (65) also found the viral shedding time was significantly shorter in asymptomatic infections (10.9 days, 95% CI: 8.3–14.3) than in symptomatic patients (19.7 days, 95%CI: 17.2–22.7). Besides, three included studies reported that the viral shedding time of asymptomatic infections was significantly shorter than that of patients with presymptomatic infection (22, 26, 27). These results indicated that patients with asymptomatic infection might recovery faster than symptomatic patients. The presence of patients with asymptomatic infection implied that the body had special mechanisms to prevent the progression of COVID-19 (31). Two included studies reported that the duration of viral shedding of asymptomatic infections or initially no-symptom patients with normal chest CT scans was shorter than that of asymptomatic infections or initially no-symptom patients with abnormal chest CT scans (54, 56). Individuals with pneumonia or lung lesions in asymptomatic infections or presymptomatic infections were more sever and hard to treat than those without pneumonia or lung lesions. However, these speculations might not be reliable because all data about the duration of viral shedding were detected by RT-PCR and a RNA testing could not distinguish whether the virus was alive or dead (66). We found that in patients with presymptomatic infection, symptoms were developed in <13 days (from diagnosis time to symptoms developed), which was 22 days in one study (19). A long time to develop symptoms indicated difficulties in controlling the COVID-19 pandemic.

Studies have shown that patients with asymptomatic infection were more common in populations of young and middle-aged individuals without underlying diseases. Some included studies of the present review reported that patients with asymptomatic infection were significantly younger than patients with presymptomatic infection (26, 27, 35, 53) or symptomatic patients (44, 47). The included studies also reported that asymptomatic infections with normal chest CT scans were significantly younger than asymptomatic infections with abnormal chest CT scans (21, 56). A meta-analysis, which included 506 patients with asymptomatic infection from 34 studies, found that the patients with normal radiology were younger than patients with abnormal radiology (*p* = 0.013) (67).

We found 65% of initial no-symptoms COVID-19 patients did not present any COVID-19-related symptom during follow-up or by end of disease course. A meta-analysis, which included 41 studies, reported that the pooled percentage of patients with presymptomatic infection among patients with no symptoms at the screening point was 48.9% (95% CI: 31.6–66.2%) (16). A systematic review which included 14 longitudinal studies reported that the proportion of asymptomatic infections among initial no-symptoms COVID-19 patients was 72.3% (68). The high proportion of patients with asymptomatic infection in initially no-symptoms COVID-19 patients found in the present meta-analysis implied the tough job of China in the later COVID-19 pandemic control.

COVID-19 should be considered among individuals with CT abnormalities even when they did not show any clinical symptoms. COVID-19 could result in lung injury even in cases without any COVID-19-related symptom. We found that the proportions of individuals with abnormal CT features in initial no-symptoms COVID-19 patients or patients with asymptomatic infection at the screening point were considerably high. A meta-analysis reported that the proportion of individuals with abnormal CT features in initial no-symptoms COVID-19 patients was 63% (95% CI: 44–78%) and that the proportion of individuals with abnormal CT features in patients with asymptomatic infection was 62% (95% CI: 38–81%) (69). Another meta-analysis found that the proportion of participants with abnormal CT features in asymptomatic infections was 62% (67). A meta-analysis found that the proportion of individuals with abnormal CT features in patients with asymptomatic infection was 47.6% (31.1–72.9%) (15). We found nearly half of initial no-symptoms COVID-19 patients and asymptomatic infections with abnormal CT features had bilateral lung abnormality. A systematic review and meta-analysis found that 41.7% of asymptomatic infections had bilateral lung involvement in the chest CT results (16). Most of the included studies reported patients with asymptomatic infection had ground-glass opacities (GGO) in their lungs. Peripheral and bilateral GGO with or without consolidation or visible intralobular lines were a typical chest CT appearance in COVID-19. However, some included studies reported that in some asymptomatic infections or presymptomatic infections without any radiological findings at the time of diagnosis and on the follow-up CT (27, 42, 54, 56, 57), which would be missed if chest CT was the only screening method.

The adjusted immune system plays an important role in determining the progression of COVID-2019 (70). The SARS-CoV-2-specific IgM and IgG yield different responses during the disease course. IgM usually wanes rapidly (71), whereas IgG usually maintains a high level for a long period (72). This phenomenon might explain the higher proportion of IgG^+^ than IgM^+^ in patients with asymptomatic infection. A meta-analysis found that the accuracy rate, sensitivity, and specificity were: (a) 0.95 (95% CI: 0.93–0.97), 0.74 (95% CI: 0.65–0.81), and 0.99 (95% CI: 0.97–1.00), respectively, for IgM and (b) 0.99 (95% CI: 0.97–0.99), 0.85 (95% CI: 0.79–0.90), and 0.99 (95% CI: 0.98–1.00), respectively, for IgG in the diagnosis of COVID-19 (73). However, IgG and IgM were reported to be seronegative till the end of disease course in some patients with asymptomatic infection in the included studies, which would be missed if anti-SARS-CoV-2 IgG/IgM testing was the only screening method.

In the present review, lymphocytopenia; leukopenia; leukocytosis; elevated CRP, LDH, ALT, Cr, D-dimer, and PCT levels; elevated ESR; and reduced albumin and hemoglobin levels in asymptomatic cases were observed. Lymphocytopenia was associated with increased COVID-19 severity (73–75). The inflammatory cytokine storm, exhaustion of T cells, and the COVID-19 infection interfering with T cell expansion were likely key factors behind the observed lymphocytopenia (76, 77). A systematic review and meta-analysis found that leukocytosis and elevated CRP were associated with poor outcomes (OR [95% CI]: 4.51 [2.53–8.04] and 11.97 [4.97–28.8], respectively), whereas leukopenia was associated with a better prognosis (OR [95% CI]: 0.56 [0.40–0.78]) (78). A significant association between leukocytosis and mortality rate in patients with COVID-19 was observed (79). In the early stage of COVID-19, CRP and LDH levels were positively correlated with lung lesions and could reflect disease severity (80–82). Elevated ALT and Cr levels in patients with asymptomatic infection indicate liver and renal injuries, respectively. The presence of liver and renal injuries were associated with progression to severe pneumonia (83, 84). The D-dimer level was commonly elevated in patients with COVID-19. D-dimer level was correlated with disease severity and was a reliable prognostic marker for in-hospital mortality in patients with COVID-19 (85, 86). The incidence of deep vein thrombosis in patients with COVID-19 was correlated with elevated D-dimer level (87). Elevated ESR, elevated PCT level, and reduced albumin and hemoglobin levels were associated with severe COVID-19 and poor outcomes (88–91).

In China, the COVID-19 pandemic has been gradually controlled. At present, the identification and management of patients with asymptomatic infection has become an urgent problem that needs to be addressed. The most likely source of asymptomatic infections is close contacts of patients who have been diagnosed or suspected. Therefore, patients with asymptomatic infection should be detected by infection source tracking investigation, close contact screening, and active detection of the target population. RT-PCR is a gold standard in the diagnosis of COVID-19. However, the false-negative rate of RT-PCR results is up to 30% (92, 93). This may result from the inappropriate or insufficient sample, inaccurate conditions of sample storage and transportation, as well as collecting the specimen too late in the disease process. A high proportion of asymptomatic cases with abnormal chest CT and laboratorial features is found in the present systematic review and meta-analysis, implying that the chest CT scan and the SARS-CoV-2-specific IgM and IgG testing can serve as effective supplementary methods to identify asymptomatic cases in the early stage of SARS-CoV-2 infection. However, the chest CT scan and the SARS-CoV-2-specific IgM and IgG testing cannot replace RT-PCR for screening in asymptomatic patients, as there are a considerable part of asymptomatic patients without radiological findings or SARS-CoV-2-specific IgG and IgM seronegative, let alone the radiation exposure risk and the impact of vaccination on antibodies.

Several limitations of our study should be considered. First, considerable heterogeneity was observed in the study, which diminished the reliability of results. Although heterogeneity decreased in the subgroup analysis, it was still high. The substantial heterogeneity across studies might be related to sample sizes, study regions, study populations, and time of data collection. Second, the chest CT scan and blood laboratory sampling were performed at different time points of infection although most of the included studies reported conducting these testing on admission. Radiological features and laboratory characteristics could have changed along with the progression of COVID-19, thereby diminishing the reliability of results. Third, the impact of false negative PCR results was not considered, which might be more likely to occur in patients with asymptomatic (94) and would underestimate the proportion of patients with asymptomatic infection. Fourth, most of the included studies were retrospective studies which might result in bias in conclusions. Fifth, we only included the studies performed in China, which limited the generalization of findings to other regions of the world. Sixth, we did not use MeSH terms in retrieving studies which might miss some related studies. Seventh, all participants were children in two studies which might confound the results.

## Conclusion

This manuscript reviewed the epidemiological, radiographical, and laboratorial characteristics of Chinese asymptomatic cases with COVID-19. We found a high proportion of asymptomatic cases with abnormal chest CT and laboratorial features. The chest CT scan and the SARS-CoV-2-specific IgM and IgG testing could serve as effective supplementary methods to identify asymptomatic cases in the early stage of SARS-CoV-2 infection. However, the chest CT scan and the SARS-CoV-2-specific IgM and IgG testing should not replace RT-PCR for screening in asymptomatic patients, because there were a considerable part of asymptomatic patients without radiological findings or SARS-CoV-2-specific IgG and IgM seronegative, let alone the radiation exposure risk and the impact of vaccination on antibodies. The combination of repeated RT-PCR, chest CT scans, and the SARS-CoV-2-specific IgM and IgG testing should be performed for those highly suspected SARS-CoV-2 infections. The specific characteristics of asymptomatic infections such as the infectiousness and outcomes of asymptomatic or presymptomatic infections with abnormal or normal findings in CT scan or laboratorial testing need to be further clarified. More longitudinal and prospective studies are needed.

## Data Availability Statement

The original contributions presented in the study are included in the article/Supplementary Material, further inquiries can be directed to the corresponding author.

## Author Contributions

HY designed the study and wrote the first draft of the manuscript. WG, YD, and HY conducted the literature search, study selection, quality assessment, and statistical analysis. WG and YD suggested improvements. All authors contributed to the final work and submission.

## Funding

This study was supported by grants from the National Natural Science Foundation of China (Grant No. 81771447).

## Conflict of Interest

The authors declare that the research was conducted in the absence of any commercial or financial relationships that could be construed as a potential conflict of interest.

## Publisher's Note

All claims expressed in this article are solely those of the authors and do not necessarily represent those of their affiliated organizations, or those of the publisher, the editors and the reviewers. Any product that may be evaluated in this article, or claim that may be made by its manufacturer, is not guaranteed or endorsed by the publisher.
