# Supplementary figures and images for: Epidemiological, Radiographical, and Laboratorial Characteristics of Chinese Asymptomatic Cases With COVID-19: A Systematic Review and Meta-Analysis
Source: Front Public Health. 2022 Mar 31;10:808471. doi: 10.3389/fpubh.2022.808471 (PMC9008196; doi:10.3389/fpubh.2022.808471)

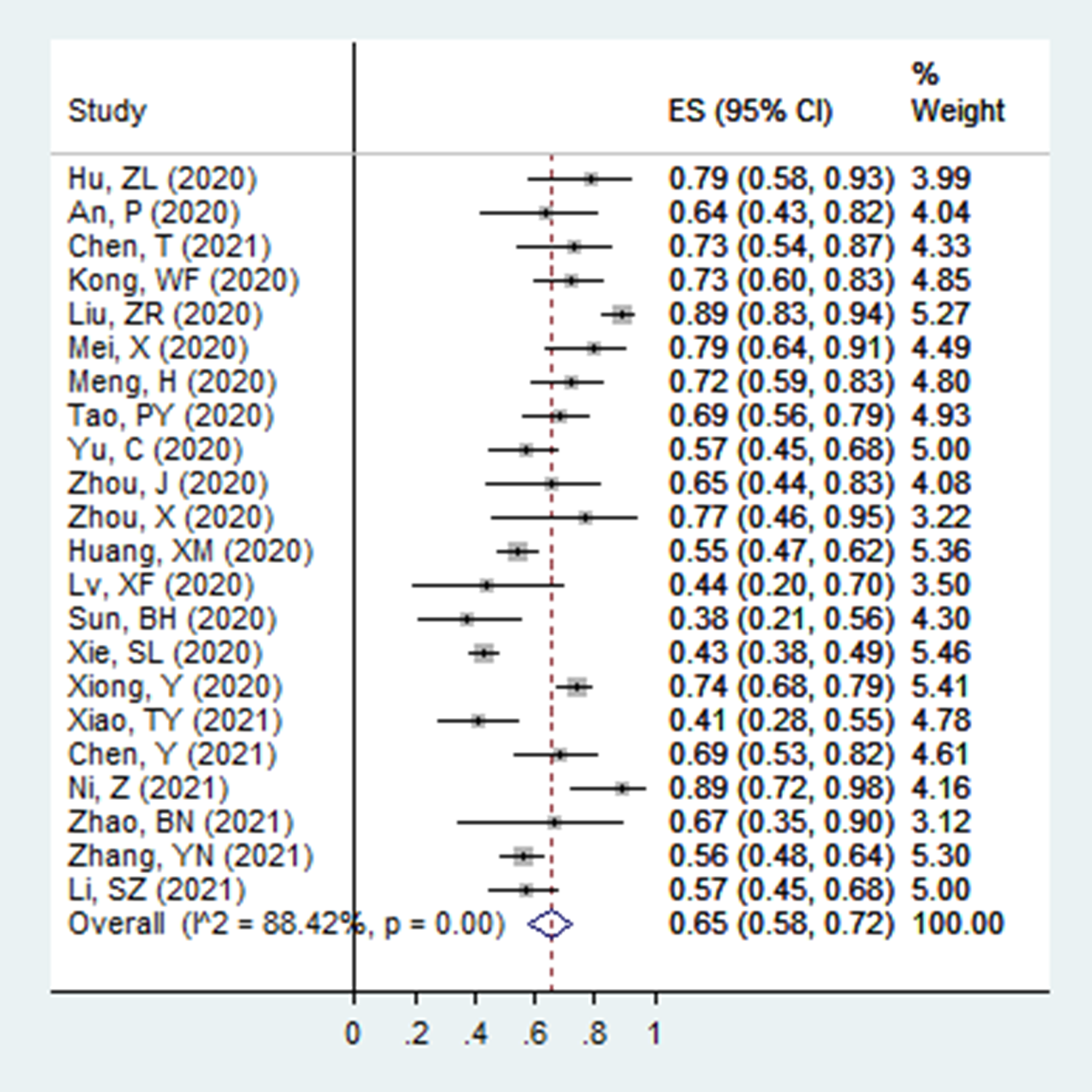

Supplement: Supplementary file 1 [file Image_1.TIF]

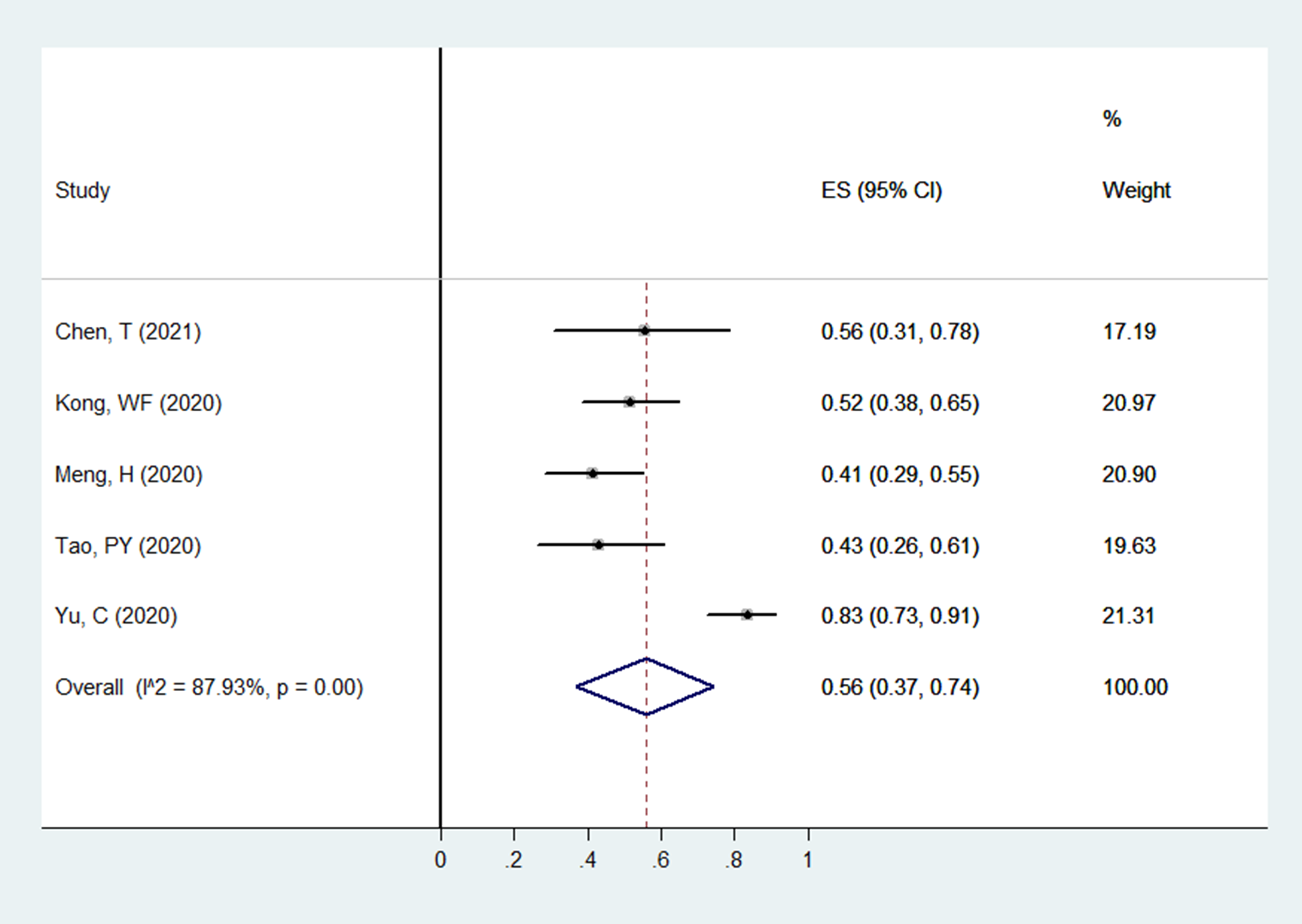

Supplement: Supplementary file 2 [file Image_2.TIF]

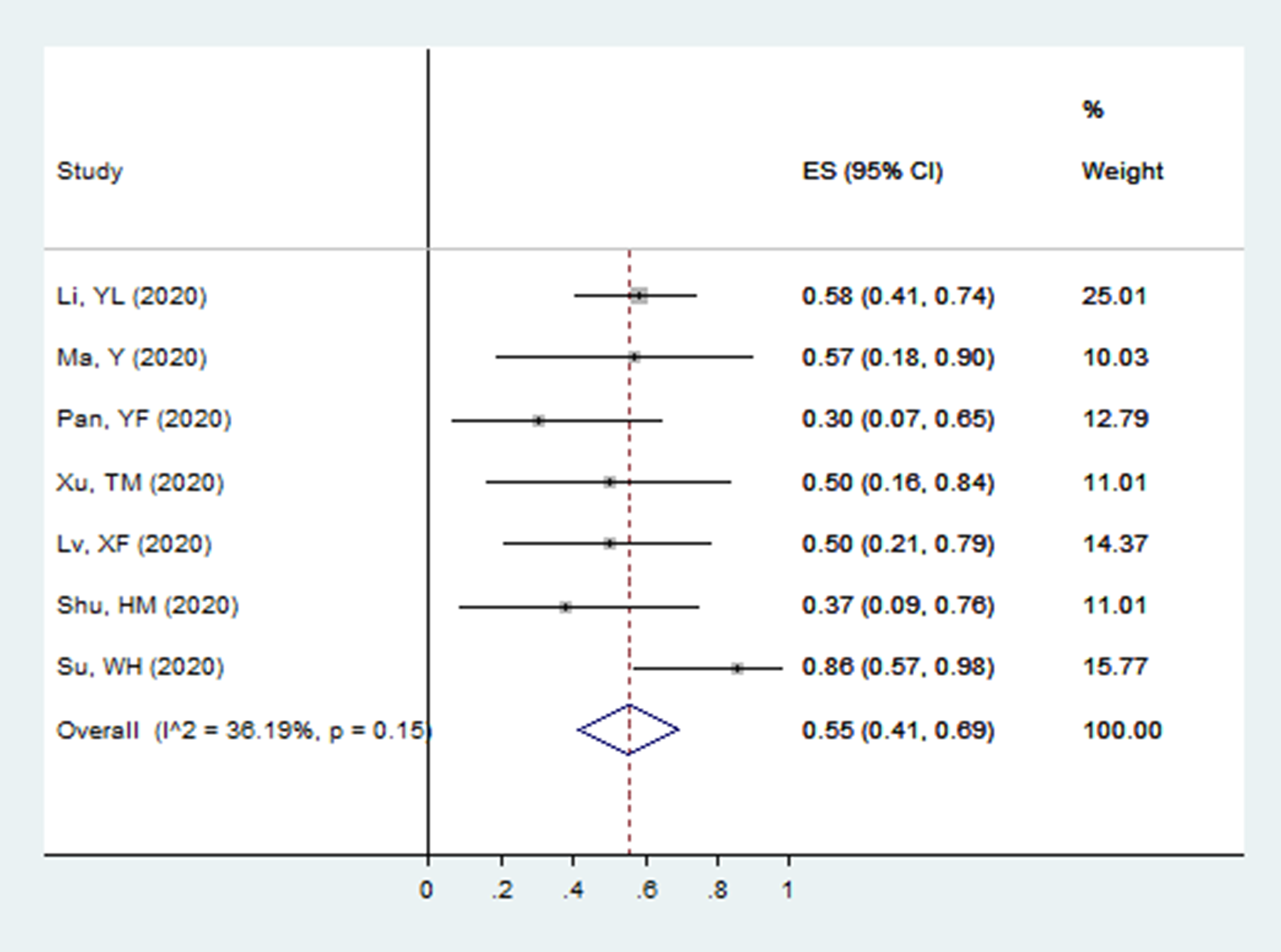

Supplement: Supplementary file 3 [file Image_3.TIF]

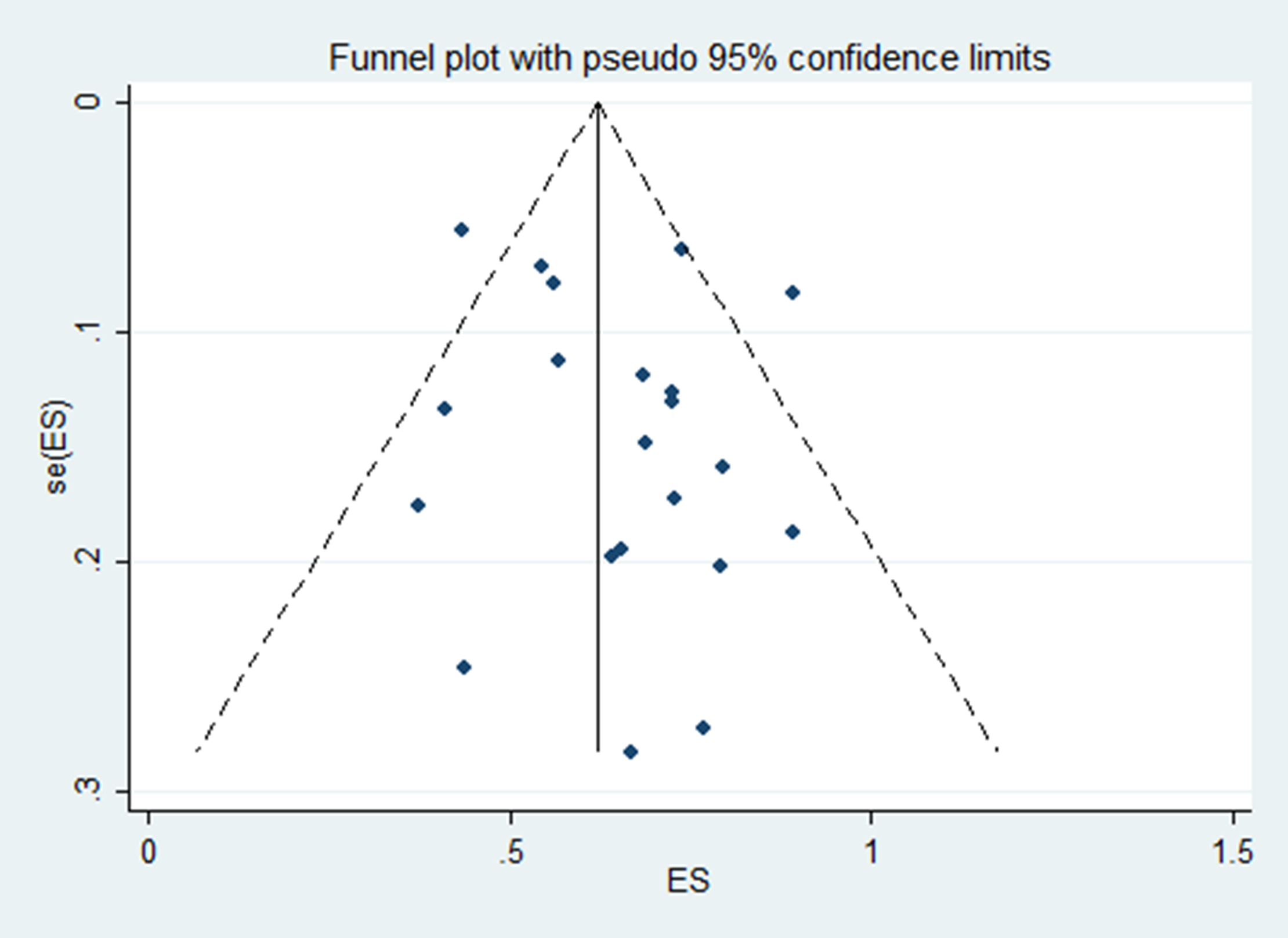

Supplement: Supplementary file 4 [file Image_4.TIF]

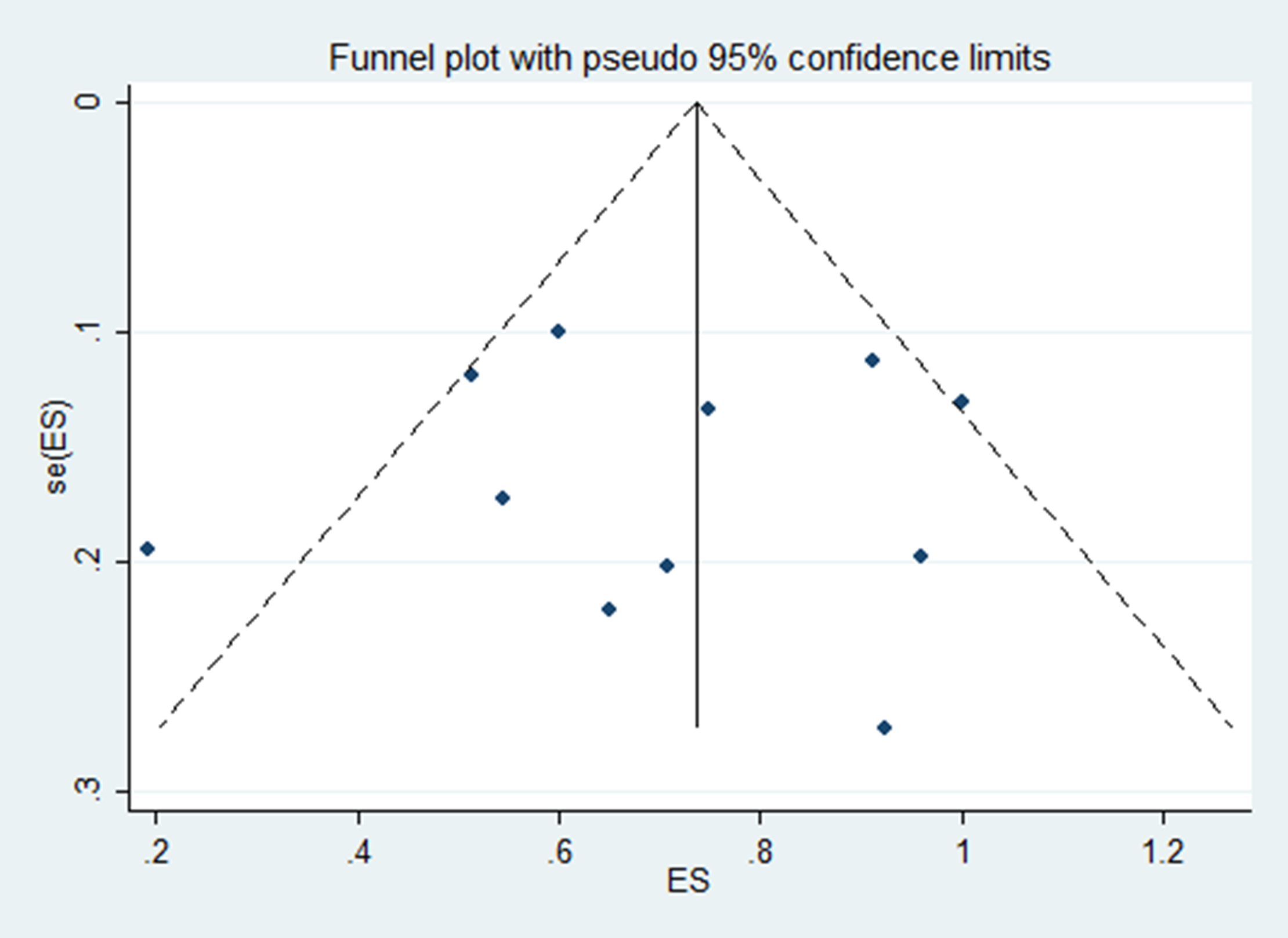

Supplement: Supplementary file 5 [file Image_5.TIF]

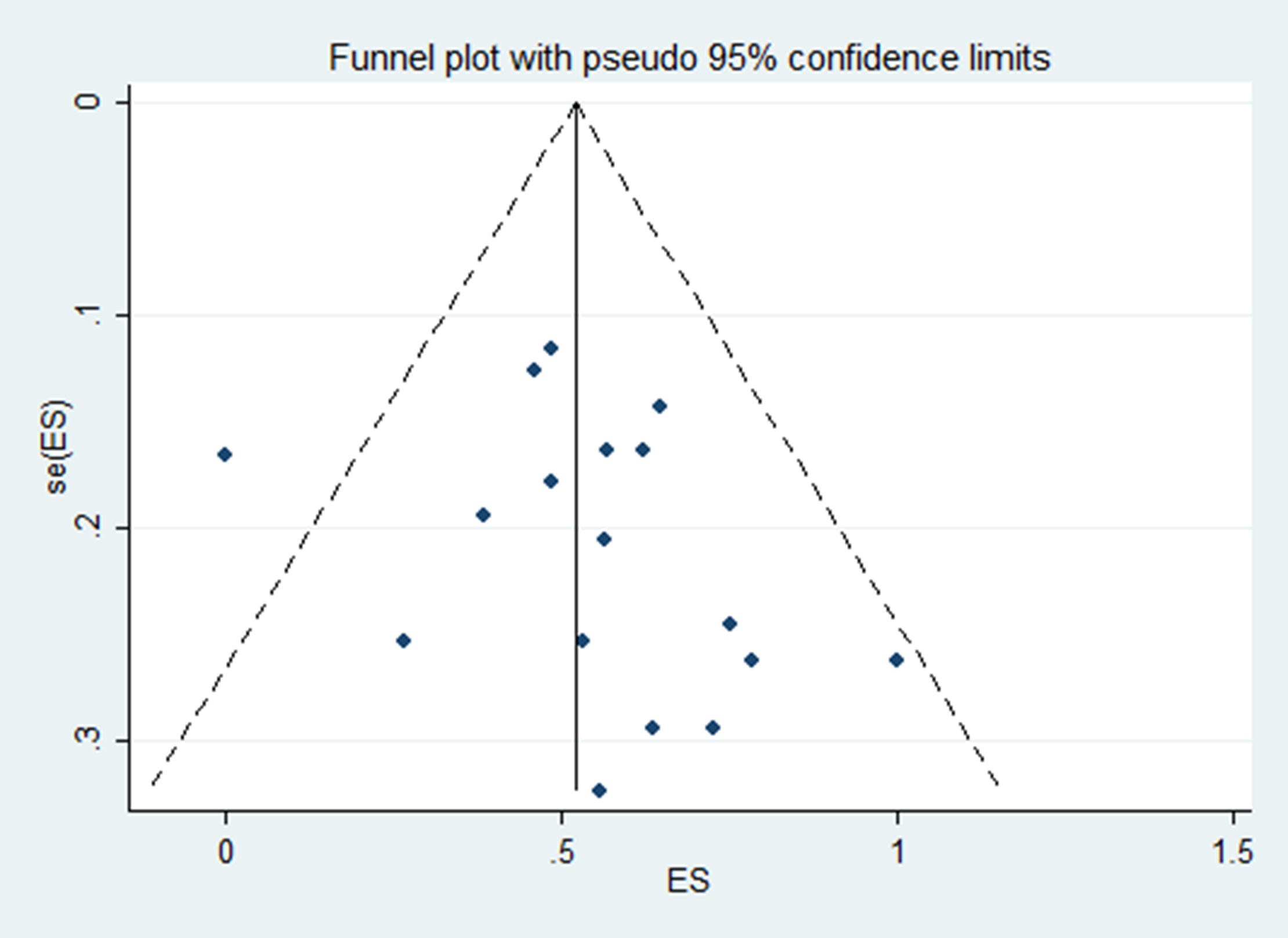

Supplement: Supplementary file 6 [file Image_6.TIF]
