# Supplementary material for: Epidemiological, Radiographical, and Laboratorial Characteristics of Chinese Asymptomatic Cases With COVID-19: A Systematic Review and Meta-Analysis
Source: Front Public Health. 2022 Mar 31;10:808471. doi: 10.3389/fpubh.2022.808471 (PMC9008196; doi:10.3389/fpubh.2022.808471)
Supplement: Supplementary file 7 [file Table_1.pdf]

Table S1: Characteristics of the included studies.

Author	Location	Study design	Survey time (2020)	Participants	Sample size	Age (year)	Male	Diagnosis	Viral shedding (d)	Symptoms developed time (d)
Hu, ZL et al	Nanjing	Retrospective	Jan 28-Feb 9	A	24	32.5	8	NAT	9.5	8.0 (6.0-9.0)
An, P et al	Xiangyang	Prospective	NA	A	25	42.2	17	NAT	NA	NA
Cai, JH et al	Shanghai	Prospective	Jan 19-Apr 30	B	21	11.8±4.3	13	NAT	14.1±6.4	/
Chen, T et al	Chongqing	Retrospective	Jan-Feb	A	33	43.2±14.3	14	NAT	NA	3.0 (2.5-7.0)
Kong, WF et al	Sichuan	Prospective	Jan 25-Mar 18	A	100	37.7±19.0	55	NAT	NA	7.0 (1.0-13.0)
Lei, Q et al	Wuhan	Retrospective	Feb 17-Apr 28	B	63	NA	52	NAT	NA	/
Li, YY et al	Tuanfeng	Retrospective	Feb-Mar	B	38	34	16	NAT	NA	/
Li, YL et al	Wuhan	Retrospective	Feb 22-Mar 8	B	74	NA	35	NAT	NA	/
Liu, ZR et al	Anhui	Prospective	NA	A	147	NA	76	NAT	NA	2.0 (1.0-5.0)
Long, QX et al	Wanzhou	Retrospective	NA	B	37	41	15	NAT	19.0	/
Ma, Y et al	Jinan	Retrospective	Jan 23-Mar 10	B	11	23	6	NAT	10.0	/
Mei, X et al	Shanghai	Retrospective	Jan 20-Mar 31	A	39	26	15	NAT	NA	NA
Meng, H et al	Wuhan	Retrospective	Jan 1-Feb 23	A	58	42.6±16.6	26	NAT	NA	3.7±2.9
Pan, YF et al	Zhengzhou	Retrospective	NA	B	26	29.5	16	NAT	NA	/
Tan, F et al	Wuhan	Retrospective	Mar 20-Apr 5	B	12	37.9	9	NAT	11.5	/
Tao, PY et al	Hunan	Retrospective	Jan 28-Feb 18	A	70	33.2±20.4	36	NAT	13.2±6.8	NA
Wang, YB et al	Chongqing	Retrospective	Jan-Mar	B	63	39.3±16.5	34	NAT	NA	/
Wu, J et al	Fengjie	Retrospective	NA	B	15	5.0-76.0	5	NAT	NA	/
Xu, TM et al	Jiangsu	Retrospective	Jan 18-Feb 26	B	15	27	10	NAT	NA	/
Yan, S et al	Beijing	Retrospective	Jan 24-Mar 29	B	11	29.3±13.0	7	NAT	NA	/
Yang, RR et al	Wuhan	Retrospective	Dec 20, 2019-Mar 8, 2020	B	48	NA	NA	NAT	NA	/

Continued Table S1.

Author	Location	Study design	Survey time (2020)	Participants	Sample size	Age (year)	Male	Diagnosis	Viral shedding (d)	Symptoms developed time (d)
Yu, C et al	Wuhan	Retrospective	Jan 17-Mar 30	A	79	60	32	NAT or antibody	NA	8.0 (4.4-11.6)
Zhou, FL et al	Wuhan	Retrospective	Mar 16-Apr 16	B	28	NA	NA	NAT or antibody	NA	/
Zhou, J et al	NA	Retrospective	Jan 20-Feb 30	A	26	37.0	10	NAT	NA	NA
Zhou, X et al	Shanghai	Retrospective	NA	A	13	51.8	6	NAT	NA	NA
Huang, XM et al	Sichuan	Retrospective	Jan 29-Jun 30	A	198	NA	NA	NAT	NA	NA
Lv, XF et al	Wuhan	Retrospective	NA	B	16	36.0±9.0	8	NAT	NA	/
Sun, BH et al	Liaoning	Retrospective	Until Mar 10	A	32	NA	12	NAT	NA	2.0 (1.0-11.0)
Wang, YF et al	Wuhan	Retrospective	Jan 1-Feb 14	B	159	45.5	66	NAT	NA	/
Xie, SL et al	Guangdong	Retrospective	Jan 14-Mar 31	A	325	35.2±19.6	164	NAT	NA	NA
Xiong, Y et al	Chongqing	Retrospective	Jan 21-Jul 31	A	242	NA	NA	NAT	NA	1.2 (0.04-11.8)
Chen, J et al	Shenzhen	Retrospective	Jan20-Mar4	A	20	8.0±5.0	7	NAT	NA	NA
Zeng, HH et al	Hunan	Retrospective	until March 30	B	37	39	13	NAT	13	/
Xiao, TY et al	Shenzhen	Retrospective	Jan 11-Apr 1	A	56	39	23	NAT	C:16.6±7.5; B: 12.1±5.8	NA
Shu, HM et al	Anhui	Retrospective	NA	B	11	53	5	NAT	NA	/
Luo, QQ et al	Wuhan	Retrospective	Jan 30-Apr 15	B	16	29.1±3.7	0	NAT	NA	/

Continued Table S1.

Author	Location	Study design	Survey time (2020)	Participants	Sample size	Age (year)	Male	Diagnosis	Viral shedding (d)	Symptoms developed time (d)
Chen, Y et al	Ningbo and Zhoushan	Prospective	Jan 21-Mar 6	A	45	46	22	NAT	C:48.00 (23.8−51.3); B: 24.00 (21.0−30.8)	2.8 (1.0–5.2)
Ni, Z et al	Shanxi	Retrospective	Until Mar 26	A	28	1-82	10	NAT	NA	NA
Zhao BN et al	Chengdu	Retrospective	Mar 10-Apr 26	A	12	18-62	5	NAT	NA	NA
Zhang YN et al	Sichuan	Retrospective	Jan 28-Mar 31	A	160	NA	84	NAT	NA	2 (1-22)
Zhang H et al	Xi'an	Retrospective	Until May 10	B	25	46	NA	NAT	14 (13-19)	/
Sun L et al	Hubei	Prospective	Mar 27-Apr 26	B	56	53.7±17.6	28	NAT	NA	/
Su WH et al	Wuhan	Retrospective	Jan 11-Feb 6	B	21	35	6	NAT	NA	/
Lu YQ et al	Chongqing	Retrospective	Jan-Jun	B	41	NA	NA	NAT	NA	/
Li SZ et al	Hubei	Retrospective	Jan-Mar	A	79	NA	32	NAT	NA	NA

A: Initial no-symptoms COVID-19 patients; B: Patients with asymptomatic infection; C: Patients with pre-symptomatic infection; NAT: Nucleic acid test; NA: Not available; /: Not applicable.
