# Supplementary material for: Epidemiological, Radiographical, and Laboratorial Characteristics of Chinese Asymptomatic Cases With COVID-19: A Systematic Review and Meta-Analysis
Source: Front Public Health. 2022 Mar 31;10:808471. doi: 10.3389/fpubh.2022.808471 (PMC9008196; doi:10.3389/fpubh.2022.808471)
Supplement: Supplementary file 8 [file Table_2.pdf]

Table S2: Quality assessment of the included studies.

Author	11-item checklist recommended by the Agency for Healthcare Research and Quality (AHRQ)											Score (Rating)
	Q1	Q2	Q3	Q4	Q5	Q6	Q7	Q8	Q9	Q10	Q11	
Hu, ZL et al	Yes	No	Yes	No	No	No	No	No	No	Yes	Yes	4 (Moderate)
An, P et al	Yes	No	Yes	No	No	No	No	No	No	Yes	Yes	4 (Moderate)
Cai, JH et al	Yes	Yes	Yes	No	No	Yes	No	No	No	Yes	Yes	6 (Moderate)
Chen, T et al	Yes	Yes	Yes	No	No	No	No	Yes	No	Yes	Yes	6 (Moderate)
Kong, WF et al	Yes	Yes	Yes	No	No	No	Yes	No	Yes	Yes	Yes	7 (Moderate)
Lei, Q et al	Yes	Yes	Yes	No	No	No	Yes	Yes	No	Yes	Yes	7 (Moderate)
Li, YY et al	Yes	No	Yes	No	No	Yes	Yes	No	Yes	Yes	Yes	7 (Moderate)
Li, YL et al	Yes	Yes	Yes	No	No	Yes	Yes	Yes	No	Yes	Yes	8 (High)
Liu, ZR et al	Yes	Yes	Yes	No	No	No	No	Yes	No	Yes	Yes	6 (Moderate)
Long, QX et al	Yes	No	Yes	No	No	No	No	No	No	Yes	Yes	4 (Moderate)
Ma, Y et al	Yes	No	Yes	No	No	No	No	No	No	Yes	Yes	4 (Moderate)
Mei, X et al	Yes	Yes	Yes	No	Yes	Yes	No	No	Yes	Yes	Yes	8 (High)
Meng, H et al	Yes	Yes	Yes	No	Yes	Yes	No	Yes	No	Yes	Yes	8 (High)
Pan, YF et al	Yes	Yes	Yes	No	No	No	Yes	No	No	Yes	Yes	6 (Moderate)
Tan, F et al	Yes	No	Yes	No	No	No	No	Yes	No	Yes	Yes	5 (Moderate)
Tao, PY et al	Yes	Yes	Yes	No	No	No	No	Yes	No	Yes	Yes	5 (Moderate)
Wang, YB et al	Yes	Yes	Yes	No	No	Yes	No	No	No	Yes	Yes	6 (Moderate)
Wu, J et al	Yes	No	Yes	No	No	No	No	No	No	Yes	Yes	4 (Moderate)
Xu, TM et al	Yes	Yes	Yes	No	Yes	Yes	No	No	No	Yes	Yes	7 (Moderate)
Yan, S et al	Yes	Yes	Yes	No	No	Yes	Yes	No	Yes	Yes	Yes	8 (High)

Continued

Author	11-item checklist recommended by the Agency for Healthcare Research and Quality (AHRQ)											Score (Rating)
	Q1	Q2	Q3	Q4	Q5	Q6	Q7	Q8	Q9	Q10	Q11	
Yang, RR et al	Yes	Yes	Yes	No	No	Yes	No	No	No	Yes	Yes	6 (Moderate)
Yu, C et al	Yes	Yes	Yes	No	No	Yes	Yes	Yes	No	Yes	Yes	8 (High)
Zhou, FL et al	Yes	Yes	Yes	No	No	No	No	Yes	No	Yes	Yes	6 (Moderate)
Zhou, J et al	Yes	No	Yes	No	No	No	No	No	No	Yes	Yes	4 (Moderate)
Zhou, X et al	Yes	No	Yes	No	No	No	No	No	No	Yes	Yes	4 (Moderate)
Huang, XM et al	Yes	No	Yes	No	No	No	No	Yes	Yes	Yes	Yes	5 (Moderate)
Lv, XF et al	Yes	Yes	Yes	No	Yes	Yes	No	No	No	Yes	Yes	7 (Moderate)
Sun, BH et al	Yes	Yes	Yes	No	No	No	No	No	No	Yes	Yes	5 (Moderate)
Wang, YF et al	Yes	No	Yes	No	No	No	No	No	No	Yes	Yes	4 (Moderate)
Xie, SL et al	Yes	Yes	Yes	No	No	Yes	No	Yes	No	No	Yes	6 (Moderate)
Xiong, Y et al	Yes	Yes	Yes	No	No	No	No	Yes	Yes	Yes	Yes	7 (Moderate)
Chen, J et al	Yes	Yes	Yes	No	No	No	No	No	No	Yes	Yes	5 (Moderate)
Zeng, HH et al	Yes	Yes	Yes	No	No	Yes	Yes	No	No	Yes	Yes	7 (Moderate)
Xiao, TY et al	Yes	Yes	Yes	No	No	No	Yes	No	Yes	Yes	Yes	7 (Moderate)
Shu, HM et al	Yes	Yes	Yes	No	No	Yes	No	Yes	No	Yes	Yes	7 (Moderate)
Luo, QQ et al	Yes	Yes	Yes	No	Yes	No	No	No	No	Yes	Yes	6 (Moderate)
Chen, Y et al	Yes	Yes	Yes	No	No	No	No	No	No	Yes	Yes	5 (Moderate)
Ni, Z et al	Yes	Yes	Yes	No	No	No	No	No	No	Yes	Yes	5 (Moderate)
Zhao BN et al	Yes	Yes	Yes	No	No	No	No	No	No	Yes	Yes	5 (Moderate)
Zhang YN et al	Yes	No	Yes	No	No	No	No	No	No	Yes	Yes	4 (Moderate)

Continued

Author	11-item checklist recommended by the Agency for Healthcare Research and Quality (AHRQ)											Score (Rating)
	Q1	Q2	Q3	Q4	Q5	Q6	Q7	Q8	Q9	Q10	Q11	
Zhang H et al	Yes	Yes	Yes	No	No	No	No	No	No	Yes	Yes	5 (Moderate)
Sun L et al	Yes	Yes	Yes	No	No	No	No	No	No	Yes	Yes	5 (Moderate)
Su WH et al	Yes	Yes	Yes	No	No	Yes	No	No	No	Yes	Yes	6 (Moderate)
Lu YQ et al	Yes	Yes	Yes	No	No	No	No	No	No	Yes	Yes	5 (Moderate)
Li SZ et al	Yes	Yes	Yes	No	No	Yes	Yes	No	No	Yes	Yes	7 (Moderate)

Q1, Define the source of information?

Q2, List inclusion and exclusion criteria?

Q3, Indicate time period used for identifying patients?

Q4, Indicate whether or not subjects were consecutive?

Q5, Indicate if evaluators of subjective components of study were masked to other aspects of the status of the participants?

Q6, Describe any quality assurance assessments?

Q7, Explain any patient exclusions from analysis?

Q8, Describe how confounding was assessed and/or controlled?

Q9, If applicable, explain how missing data were handled in the analysis?

Q10, Summarize patient response rates and completeness of data collection?

Q11, Clarify what follow-up, if any, was expected and the proportion of missing data?
